# Ternary Graphene Oxide and Titania Nanoparticles-Based Nanocomposites for Dye Photocatalytic Degradation: A Review

**DOI:** 10.3390/ma17010135

**Published:** 2023-12-27

**Authors:** Jessica Campos-Delgado, María Eugenia Mendoza

**Affiliations:** Instituto de Física, Benemérita Universidad Autónoma de Puebla, Av. San Claudio esq. 18 Sur, Puebla 72570, Mexico; emendoza@ifuap.buap.mx

**Keywords:** graphene oxide, titania, photocatalysis, dye, ternary nanocomposites

## Abstract

Advanced oxidation processes stand as green alternatives for the decontamination of waste waters. Photocatalysis is an advanced oxidation process in which a semiconductor material absorbs photon energy and triggers redox reactions capable of degrading organic pollutants. Titanium dioxide (TiO_2_, titania) represents one of the most popular choices of photocatalytic materials, however the UV-activation of its anatase phase and its high charge recombination rate decrease its photocatalytic activity and weaken its potential. Graphene oxide is a 2D carbon nanomaterial consisting of exfoliated sheets of hexagonally arranged carbons decorated with oxygen- and hydrogen- functional groups. Composite nanomaterials consisting of titania nanoparticles and graphene oxide have proven to enhance the photocatalytic activity of pure TiO_2_. In this review, we present a thorough literature review of ternary nanocomposites based on synthesized or commercial titania nanoparticles and GO (or reduced GO) particularly used for the photodegradation of dyes. GO/TiO_2_ has been enriched primarily with metals, semiconductors and magnetic nanomaterials, proving a superior dye degradation performance and reusability compared to bare TiO_2_. Ongoing challenges and perspectives are outlined.

## 1. Introduction

Water availability for human consumption is threatened, paradoxically, by human activities. Facing the rapidly growing menace of water scarcity and water quality, treatment of wastewater stands as an imminent measure for health and environmental issues. Wastewater might contain residues of human waste, food and chemicals, among others. According to the United Nations, 90% of sewage in developing countries is discharged untreated directly into water bodies and an estimated 300–400 megatonnes of waste are discharged by industries into water bodies every year [1]. Industrial effluents carry residues from the processes performed in the different activities (food, textile, petrochemical, chemical, pharmaceutical, steel, automotive, etc.), hence, industrial wastewaters can be highly polluted with heavy metals, solvents like benzene or chloroform, synthetic dyes, organic compounds that can be endocrine disruptors, drugs, among others [2]. Traditional treatments of wastewater include physical, chemical, and biological approaches. Physical methods consist of sieves and filters along with sedimentation processes that could trap/separate macroscopic and micrometer-sized solid particles. Biological methods involve microorganisms (bacteria, yeast, algae) to digest the pollutants and separate them. Chemical techniques rely on separation and sedimentation by chemical reactions, adsorption and ion exchange [3]. Although the biotechnological processes represent an economic and versatile alternative, the heterogeneity of the pollutants in wastewaters makes the design of removal difficult due to their wide chemical spectrum, biological processes result thus in incomplete removal [4]. Since the 1980s, new alternatives for the decontamination of water have arisen, namely the so-called advanced oxidation processes (AOPs). This review focuses on photocatalysis, an AOP used for the photodegradation of dyes enabled by titania nanoparticles coupled with graphene oxide and a third component (metal, semiconductor or magnetic material), forming ternary nanocomposites; to the best of our knowledge, this is the first document of its kind. We have excluded from this revision nanosystems based on other nanoforms of TiO_2_ (nanotubes, nanorods, nanofilms) and we have limited ourselves to research based on powdered materials, excluding membrane-technology or other forms of photocatalytic films.

### 1.1. AOP (Advanced Oxidation Processes)

Advanced oxidation processes represent a viable route towards the degradation of heavily toxic, recalcitrant contaminants; these processes rely on the in situ formation of strong oxidants (e.g., hydroxyl and sulfate radicals) for water purification. These methods stand as an alternative to the traditional wastewater treatments when proven ineffective as more rigorous standards are set by environmental regulations [5]. AOPs can be catalogued into (a) UV–hydrogen peroxide processes, (b) Fenton and photo-Fenton, (c) ozone-based processes, (d) photocatalysis and (e) sonolysis [6]. Here, we will focus on photocatalytic reactions towards the degradation of dyes.

### 1.2. Photocatalysis

**Figure 1:**
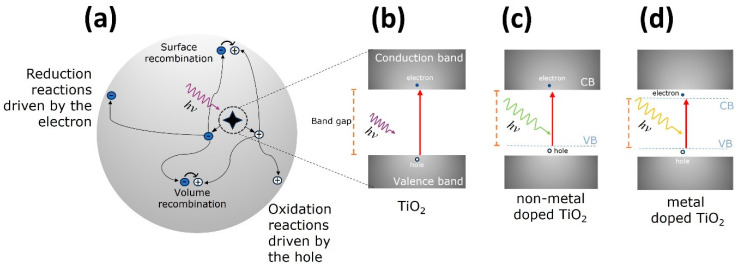
(**a**) Schematics of the photocatalytic process on a titania nanoparticle illustrating the possible paths for the e-h pair generated [7]; (**b**) photoabsorption process where an incident photon of *hν* energy triggers the jump of an electron (full circle) from the valence band (VB) to the conduction band (CB), leaving behind a hole (empty circle) on the VB; band gap engineering of TiO_2_ to reduce and tune its band gap towards the visible, (**c**) non-metal doping and (**d**) metal doping.

The photocatalytic activity of semiconductors is triggered by the absorption of a photon with sufficient energy to allow a transition of an electron from the filled valence band (VB) to the empty conduction band (CB), leaving behind a hole in the VB, thus an electron-hole (e-h) pair is formed, as depicted in Figure 1a,b.

The charge separation events happen within the volume or at the surface of the semiconductor. There are several paths that the e-h pair can follow: (1) recombination at the volume or at the surface, where the electron falls back to the hole releasing heat; (2) the electron migrates to the surface and promotes the reduction of an electron acceptor specie (usually oxygen) pre-adsorbed to the semiconductor surface; (3) the hole migrates to the surface where an electron from a donor specie can combine with the surface hole, oxidizing the donor specie; an illustrative diagram of these possibilities is presented in Figure 1a. The probability and rate of the charge transfer processes for electrons and holes depend on the width of the band gap and the redox potential levels of the adsorbate species [7].

The high proportion of e-h pairs following path (1) described previously accounts for another important setback of semiconductors: only 10% of the photogenerated e-h pairs could be used for semiconductor photocatalysis. Efforts to reduce the recombination of e-h pairs represent a well-known strategy to increase photocatalytic performance [8].

Based on the physical state of the reactant and the photocatalytic semiconductor, photocatalysis can be homogeneous (both in the same state) or heterogenous (reactant in one state and the semiconductor in a different one).

### 1.3. Dyes

Dyes are compounds that provide pigmentation to fabric, leather, paper, paint or any colorable material. Through time, human communities extracted pigments from nature, either from minerals or from plants and insects [9]. At present, the dye industry relies on chemistry to synthesize the massive amount of dye material demanded by the textile, paper, tannery, cosmetic and paint industries, among others. The textile industry prevails as the number one consumer of dyestuff at approximately 10,000 tonnes per year worldwide [10]. Large quantities of water are required for textile processing, dyeing and printing; water consumption for dyeing varies from 30 to 50 L per kg of cloth depending on the type of dye used [11]. Textile processes require mixtures of chemicals, dyestuff and water and it is estimated that only 10% of dye and chemicals are absorbed by fabrics. The remaining dye effluent is discharged, often untreated, into the environment [12]. One way to classify dyes is based on their chemical structure, as acid, basic, direct, azo, reactive, mordant, vat, disperse, and sulfur dyes, with azo dyes being the most used currently [13]. Another way to classify them is using their molecular charge upon dissociation in aqueous-based applications, as cationic and anionic dyes. Cationic dyes contain cationic functional groups that can dissociate into positively charged ions in an aqueous solution (methylene blue, rhodamine B, malachite green, crystal violet), most cations are N^+^ ions. Anionic dyes cover direct, acid, and reactive dyes—these dyes contain anionic functional groups, i.e., sulfonic or carboxylic acid groups, these functional groups are water soluble and can successfully interact with photocatalysts with hydrophilic surfaces (acid orange 7, methyl orange) [13].

Synthetic dyes are designed to last, they are intended to be highly stable and to withstand degradation upon contact with water, detergents, washing agents, light exposure and heat [10]. These complex organic structures are therefore persistent upon traditional wastewater treatments, moreover, of higher concern are the by-products generated after these processes. Even though reactive textile dyes can be decolorized under anaerobic conditions due to reduction of the azo bond, the resultant aromatic amines resist further degradation and may be toxic or genotoxic [14]. Alves de Lima and co-workers [15] reported that the effluent discharge of a textile azo-dye processing plant was used as input in a drinking water treatment plant in the Cristais River in Brazil and this drinking water tested positive for mutagenic and carcinogenic essays. The chemical degradation of dyes is a very complex process, their complete mineralization must be the goal, this is, to decompose up to obtain CO_2_, H_2_O, N_2_, [NO_3_]^−^, [SO_2_]^2−^, etc., however, considering different reports, the following possibilities of “degradation” have been proposed: photodecolourization (reversible photooxidation or photoreduction); photodegradation (decomposition to stable compounds) and photomineralization (complete decomposition). Ajmal et al. [16] have provided a comprehensive review on the mechanisms of dye photocatalytic degradation by TiO_2_ and the interested reader is advised to revise it. As Katheresan and co-authors [10] state, the answer is not to turn back to natural dyes since the substances used to make sure the natural dyes bond to fabrics (mordants) are toxic and even more dangerous than synthetic dyes. A viable and effective solution for dye degradation from wastewaters is urgently needed.

### 1.4. Graphene Oxide (GO)

**Figure 2:**
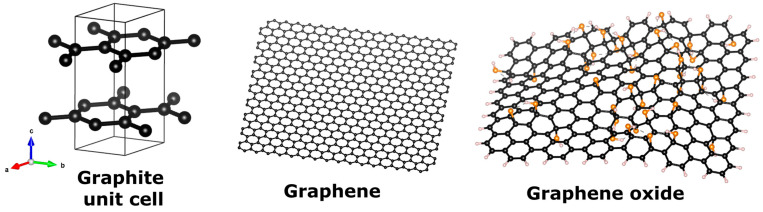
Atomistic models (simulated using VESTA) of unit cell of graphite, graphene, and graphene oxide. Black, orange and white atoms represent carbon, oxygen and hydrogen, respectively. Coordinates sources: (a) COD database code 9011577, (b,c) CSIRO data access portal.

Graphene oxide is a 2D carbon nanomaterial. Graphite, its parent material, possesses a hexagonal crystalline structure (space group P6_3_/mmc) where the sp^2^ hybridized carbons form in-plane covalently bonded hexagonal rings, as can be visualized in Figure 2 where the unit cell of graphite is depicted. The periodic repetition of these unit cells in the three dimensions results in continuous layers of a honey-comb carbon pattern, piled up following a certain stacking order (AB or Bernal stacking), thus forming the graphite crystal. The layers (called graphene) are weakly bonded to each other by van der Waals forces, whose strength is of the order of 10^−20^ J as measured by raw bimodal AFM methodology by Chiou and co-authors [17]. When the layers of graphite are separated (exfoliated), single-layer graphene can be obtained, in fact, the very first observation of graphene was from detached layers of graphite crystal [18]. Graphene oxide is composed of exfoliated graphene sheets, functionalized with oxygen- and hydrogen- containing groups; the primary objective of these functional groups is to render the nanomaterial hydrophilic since its graphene counterpart is non-soluble in water. The structure of graphene consists of continuous monolayers of sp^2^ hybridized carbon atoms, arranged in a hexagonal pattern (see Figure 2). It is considered a wonder material due to its light weight, high electron mobility (up to 200,000 cm^2^/Vs [19]), transparency, and strength. In graphene oxide (GO), sp^3^ hybridization is also present and allows for in-plane anchoring of hydroxyl (OH), carboxyl (COOH), carbonyl (C=O) and/or epoxide (C-O-C) groups; the exact amount and proportion of these functional groups along with in-plane vacancies and defects is difficult to determine and varies among samples [20], see Figure 2 for a representative diagram of GO. Unambiguously, all graphene oxide samples contain disruptions of the continuous hexagonal network resulting in diminished electrical (electron mobilities in the range 365–5000 cm^2^/Vs [21]), mechanical and thermal capacities [22]. The varying ratio of sp^2^- to sp^3^-bonded carbons and the amount of oxygen functional groups account for tunability of its bandgap and therefore GO behavior can switch from insulator to semi-metal to semiconductor [23]. Lundie and co-authors [24] have demonstrated theoretically that by controlling the reduction of graphene oxide, the energy gap decreases with the number of O adatoms removed, tuning through the ranges of UV, visible and IR light, evidencing a tunable optical response. Graphene oxide has been tested as photocatalyst for the reduction of resazurin under UV light [25] and for the photocatalytic conversion of CO_2_ to methanol under visible radiation [26]. This optical response has proven to be of paramount importance when combined with titania for the red-shifted photocatalytic response of the system due to synergetic effects between the materials.

The most common route for the synthesis of GO is the top-down approach by chemical exfoliation of graphite, moreover, this method allows for its large-scale production; another less-established alternative is the electrochemical exfoliation [20]. Chemical exfoliation consists of expanding and oxidizing graphite by strong acidic treatments, followed by thermal or mechanical (ultrasonic) exfoliation of the layers. At present, the most popular synthesis methodology is referred to as the Hummers’ method, established by William S. Hummers Jr. and Richard E. Offeman [27] in 1958. In their methodology, the oxidation of graphite is accomplished by exposing graphite to a mixture of sulfuric acid, sodium nitrate and potassium permanganate. Later changes in this method lead to what is known as the “modified Hummers’ method” [28] and “improved Hummers’ method” [29]. In the modified Hummers’ method, some groups proposed variations like the amount of KMnO_4_ and the proportion of the reactants, while the improved Hummers’ method sought higher performance of the method and lower pollution. Briefly, the improved process consists of three critical steps: H_2_SO_4_ intercalation and boric acid stabilized K_2_FeO_4_/KMnO_4_ pre-oxidation at low temperature, deep oxidation with secondary feeding of KMnO_4_ at middle temperature and hydrolysis and exfoliation of pristine graphite oxide into GO after the addition of H_2_O [20].

### 1.5. rGO (Reduced Graphene Oxide)

Efforts to revert the chemical functionalization of graphene oxide and partially recover the superior electrical and mechanical properties of graphene have resulted in research focused on reduction mechanisms via thermal, electrical, or chemical routes [30]. Heat treatments at 300–350 °C under air have proven to reduce GO; cyclic voltammetry, UV and solar photo reductions have also been reported while the chemical approach using sodium borohydride, ascorbic acid or hydrazine is the most widespread [20]. rGO possesses different properties when compared to GO since oxygen and hydrogen functional groups have been partially removed. The extent to which these functional groups are removed and the basal planes are restored is not easy to determine and can vary from one reduction method to another. However, it is true that the totality of functional groups is not expected to be removed since rGO is a water-soluble material, hence the remaining oxygen and hydrogen functional groups render it hydrophilic. The differences between both materials will depend on the GO starting material (size, degree of defects, proportion of hydroxyl (OH), carboxyl (COOH), carbonyl (C=O) and/or epoxide (C-O-C) functional groups) and its reduction method (chemical, thermal, electrical, solar). Techniques that could shine light onto the characteristics of rGO compared to GO include Raman spectroscopy, FTIR and XPS among others.

### 1.6. TiO_2_

**Figure 3:**
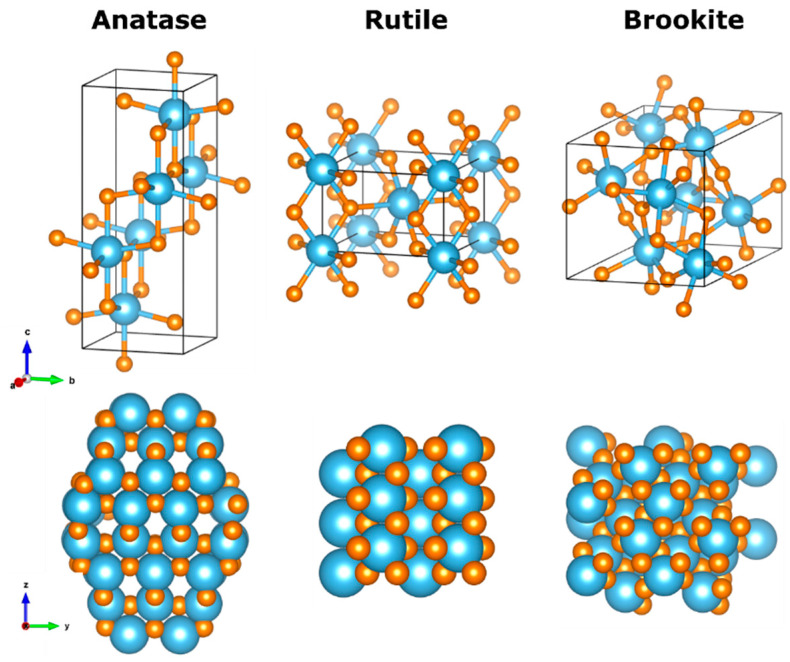
Atomistic models (simulated using VESTA) of TiO_2_ polymorphs: anatase, rutile and brookite phase titania. **Top** panels represent the unit cells of the structures while bottom panels are views of the crystals along the *x*-axis. Blue and orange atoms represent titanium and oxygen, respectively. Coordinates sources: top panels are from the COD database (anatase code 1526931, rutile code 1530150, brookite code 8104269), **bottom** panels (anatase adapted from [40], rutile and brookite openmopac.net).

Titanium dioxide or titania is one of the most popular metallic oxide semiconductors. It is non-toxic, abundant, and economically accessible. Crystalline titania can exist in three polymorphs in nature: anatase, rutile and brookite; schematic representations of the crystal unit cells as well as portions of these crystals can be found in Figure 3. Anatase and rutile are tetragonal crystals with cell parameters a = b = 0.377 nm, c = 0.943 nm for anatase [31], space group I4_1_/amd; whereas the unit cell parameters for rutile phase are a = b = 0.459 nm, c = 0.296 nm [32], space group P4_2_/mnm, while brookite crystallizes in the orthorhombic system with unit cell parameters a = 0.918 nm, b = 0.544 nm, c = 0.514 nm [33], space group Pbca. In the bulk and thin films, the anatase phase is stable up to 600 °C, at higher temperature it transforms into rutile [34]; the other eight crystalline phases have been experimentally observed and studied [35]. Of paramount importance is the capacity of TiO_2_ to arrange in nanostructured forms, i.e., nanoparticles, nanotubes, nanofilms; these nanostructures enable the dramatic increase in surface to volume ratio, therefore allowing the exploitation of the vast quantity of surface available for photocatalytic reactions [36]. Regarding TiO_2_ nanoparticles, there are reports that conclude that small spheric nanoparticles are amorphous (<5 nm), while larger faceted nanoparticles favor the anatase crystalline phase [37]. For uses in photocatalysis, the most studied materials are anatase and rutile; brookite has slowly gained attention but its use is not common yet [38]. Anatase has a higher surface area, hence larger adsorption capacities than rutile and it exhibits lower recombination rates [39]. Anatase possesses a band gap of 3.2 eV, corresponding to 384 nm, making the harvesting of solar radiation for photocatalytic activation very inefficient; rutile’s band gap, however, is slightly narrower compared to anatase, falling in the visible (3.02 eV, 410 nm). Band gap engineering through doping, surface modification or coupling with narrower band gap semiconductors enlarges the photocatalytic efficiency of anatase, however the integrity of the crystal structure should be maintained while changing its electronic structure.

Doping with metals or non-metals has been widely used to tune the optical band gap of the TiO_2_ for catalytic purposes [41]. Non-metal doping (including nitrogen, sulfur, and carbon) promotes the formation of impurity energy levels above the valence band, narrowing the energy band gap allowing for visible light activation [42], see Figure 1c. Regarding metal doping, noble (Ag, Au, Pt), transition metal ions (Ni, Cr, Fe, Zn) and rare-earths have been explored. Noble metals are resistant to photocorrosion and oxidation, moreover, the noble metal acts as electron scavengers in charge separations and as visible light activators. Transition metals cause a red shift of the TiO_2_ band gap, probably due to the overlapping of conduction band Ti(3d) with d levels of the transition metals which allow absorption of light into the visible region. However, doping of transition metals ions may also reduce the quantum efficiency because it may act as recombination sites for the photogenerated charge carriers [39]. Metal doping of the TiO_2_ crystal lattice leads to the formation of new energy states below the conduction band and above the valence band, and these new energy states act as charge-trapping sites that improve the charge-separation efficiency [43], as depicted in Figure 1d.

There are several bottom-up chemical techniques to synthesize TiO_2_ nanoparticles, among the most used we can find: hydrolysis, sol-gel, and solvothermal/hydrothermal methods. Hydrolysis usually involves TiCl_4_ and H_2_O as reactants yielding TiO_2_ and HCl, the reaction can be carried out at room temperature and nanosized polycrystalline anatase TiO_2_ nanoparticles are produced [44]. The sol-gel method generally consists of three steps. In the first step, a metal alkoxide or a metal salt is hydrolyzed in water/ethanol solution to form a metal hydroxide. The metal hydroxide then undergoes condensation to create metal-O-metal bridges, colloidal particles or sol is formed when sufficient metal-O-metal bridges are created locally. In the second step, colloidal particles link together to create a 3D network at the nanoscale (gel). In the third step, the nanoparticles are dried by heating, freeze drying or supercritical drying [45]. In this case, an organic titanium alkoxide (titanium tetra-n-butoxide or titanium tetraisopropoxide) is used, it is dissolved in an alcohol solvent (ethanol, 2-propanol, 1-pentanol, etc.) and then added to water, promoting the formation of Ti-O-Ti chains, and a TiO_2_ sol [46]. Removal of the solvent follows and a common step is a calcination at 400–500 °C under air for several hours to promote crystallization. The size for the TiO_2_ particles depends on the experimental parameters: molar ratio between precursor and water, initial pH, reaction time, presence of external ionic species, and temperature [35]. Another method consists of one-pot synthesis in which sealed stainless steel autoclaves with Teflon liners are used to control the temperature and pressure of the system, when the reactions are carried out in non-aqueous solvents it is called solvothermal and hydrothermal when water is the solvent. Experimental parameters can be tuned to attain the desired TiO_2_ properties: temperature, duration, pressure (percentage fill), solvent, pH of solution, mineralizer, surfactant, type of acid/base, calcination temperature, and the type of titanium precursor [47]. Common precursors are titanium butoxide (tetrabutyl titanate), titanium tetrachloride and titanium isopropoxide.

### 1.7. Degussa P25

Degussa P25 is a mixed-phase TiO_2_ nanomaterial that exhibits higher photocatalytic activity than its pure anatase and rutile titania counterparts [48]. This material has become a global standard in photocatalytic experiments; a Scopus search of the words “P25” and “photocatalysis” yielded more than 4300 research articles with these words within the title, abstract and keywords, evidencing the widespread use of this material in photocatalysis nowadays. The mixed-phase titania material consists of mixtures of pure crystalline anatase and pure crystalline rutile nanoparticles in proportions of 70%–30%, 80%–20% or concentrations in between these values [48,49,50,51]. The morphology of P25 consists of anatase and rutile titania nanoparticles co-existing separately within the sample [48], with varying proportions among different lots [42] or even within the same sample [49]. While the nanoparticles sizes reported are not consistent, the values are below 100 nm and usually the anatase nanoparticles are smaller than the rutile crystallites [48,50,51]. Hurum and co-workers [52] attribute the high catalytic activity of mixed-phase TiO_2_ catalysts largely to the synergistic activation of the rutile phase by anatase. The rutile phase extends the photoactive range into the visible, harvesting more light, and electron transfer from rutile to anatase trapping sites prevents charge recombination.

## 2. Binary Nanocomposites GO/TiO_2_

As mentioned above, non-metal doping with carbon is a sought-after alternative to modify the band gap of anatase nanoparticles; moreover, many research groups have explored the photocatalytic activity of composites based on TiO_2_ combined with carbonaceous nanomaterials. As Leary et al. [53] summarize, the carbon nanomaterials studied comprehend activated carbon, carbon nanotubes, C_60_, graphene and graphene oxide. A more recent review on carbonaceous materials and TiO_2_ composites is provided by Khalid and co-authors [54], while Purabgola and co-authors [55] prepared a literature review on graphene-based TiO_2_ composites. Khalid and co-authors explained the synergy of TiO_2_-carbonaceous materials based on three carbon-promoted mechanisms: (i) higher adsorption of pollutants, increasing their concentration on the vicinity of the photocatalyst, (ii) absorption on the visible range attributed to titania C-doping which narrows the band gap, and (iii) enhancement of charge separation since carbon materials enable the dissipation of electrons [54], particularly, graphene’s zero bandgap and high electron mobility allows the flow of electrons, depressing the e-h recombination [55].

Regarding the methods to synthesize these binary composites, the most straightforward is mixing both components [56], often called impregnation. This is carried out in solution, assisted by magnetic stirring or ultrasound; as highlighted by Leary [44], this method is both praised (due to its simplicity) and criticized (due to the low level of interaction between the components). Other methods involve the in situ synthesis of TiO_2_ using the above discussed methods (sol-gel [57], solvothermal/hydrothermal [58,59]) in the presence of an aqueous solution of previously prepared GO (most reports synthesize GO using the Hummers’ method or one of its variants). These wet-chemistry methods also promote the reduction of graphene oxide during the preparation of the composites, some authors intentionally add steps during the synthesis to reduce GO. Many groups submit the dry-synthesized composite powders to thermal treatments (calcination) to promote crystallization at 400–500 °C under air or Ar for several hours.

## 3. Ternary Nanocomposites GO/TiO_2_/X

As mentioned earlier, band gap engineering and overcoming the high rate of e-h pairs recombination can be tackled by synergistic mechanisms among TiO_2_, GO and third components; our literature review yielded studies that can classify such third constituents as metals, semiconductors and magnetic materials; isolated studies of other compounds are also mentioned. The methodology for the exposure of dye-polluted water to these ternary photocatalytic composites consists of adding certain amounts of the photocatalysts to a dye solution and stirring in the dark for 30–60 min to achieve adsorption/desorption equilibrium, then the dye solution with photocatalysts is exposed to the chosen type of radiation (UV, solar, visible) and sampling is performed at regular time intervals to monitor the UV-Vis response of the dye in order to quantify it. Results of photocatalytic experiments are primarily reported calculating the efficiency of degradation, as (C_0_ − C_t_)/C_0_ × 100, where C_0_ is the initial concentration and C_t_ is the concentration at time t after irradiation.

### 3.1. X = Metals

**Figure 4:**
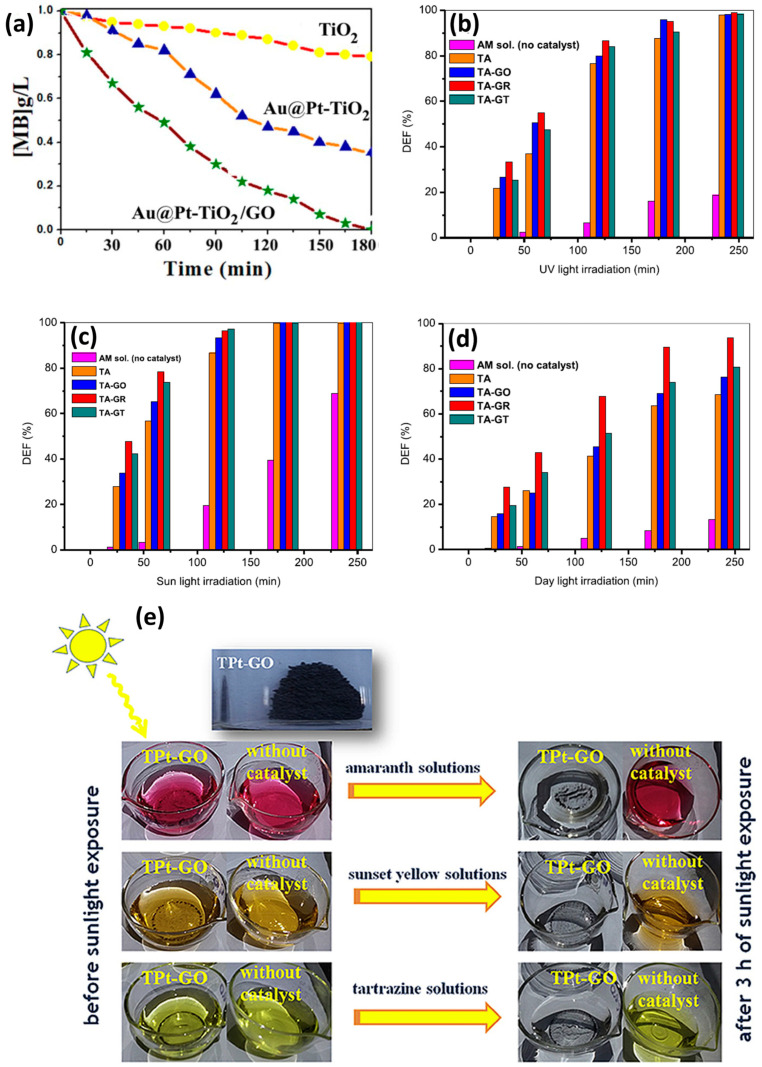
(**a**) Degradation of methylene blue (MB) by ternary composite Au@Pt-TiO_2_/GO under UV radiation (reproduced from IOP Publishing under the Creative Commons Attribution 4.0 license from [60]); (**b**–**d**) amaranth dye degradation by TiO_2_-Ag (TA) composite, and graphene, TiO_2_ and Ag ternary composites (TA-GO, TA-GR, TA-GT) under UV radiation, intense sunlight and day light, respectively, *y*-axis label DEF stands for “photodegradation efficiency” (reproduced with permission from [66], Elsevier, license 5659520835319); (**e**) visual evidence of photodegradation of amaranth, sunset yellow and tartrazine dye solutions exposed to GO/Pt/TiO_2_ ternary composites and solar irradiation (3 h) (reproduced from [61]. Copyright © 2017 Elsevier Masson SAS. All rights reserved).

The combination of GO/TiO_2_ with metals for the photodegradation of dyes involves the co-deposition of metallic nanoparticles on the surface of graphene; noble metals are the most explored (Pt, Ag, Au). Table 1 summarizes the articles found in the literature for these types of nanocomposites. Important parameters are included, such as dye degraded, type of metal added, synthesis process, degradation time, radiation type used, and catalyst efficiency of the ternary composites, moreover, the degradation efficiency of TiO_2_ (or binary composites) is included for the sake of comparison. Ben Saber and co-workers [60] tested the effect of the ternary system Au@Pt-TiO_2_/GO against pure TiO_2_ and Au@Pt-TiO_2_ composites for the degradation of methylene blue under UV radiation, their results, pictured in Figure 4a, evidence that the ternary nanocomposite outperforms the other compounds for the degradation of the dye.

**Table 1:** Summary of literature review for the photocatalytic degradation of dyes using ternary composites of GO, metal and titania (TiO_2_) nanoparticles.

Dye Degraded	Catalyst		Catalyst Efficiency		Degradation Time	Radiation Type	Reference
	Metal	Composite Synthesis Process	Ternary Composite	TiO_2_			
Methylene blue	Au-Pt	Chemical and thermal	100%	20%	3 h	UV-Vis	[60]
Amaranth	Pt	Chemical, mixed by sonication	85.6% + 99.56% -	* 75.61% + * 99.99% -	3 h	UV Solar	[61]
Sunset yellow			77.78% + 99.15% -	* 67.87% + * 98.67% -			
Tartrazine			65.32% + 96.23% -	* 58.74% + * 96.1% -			
Acid orange 7	Pt	Chemical and hydrothermal	99.1%	# 45.06%	6 h	Solar	[62]
Rhodamine B	Pt	Chemical, microwave assisted	60% + 30% ++	WD	150 min	UV Visible	[63]
Crystal violet	Ce Fe	Sonochemical	54.2% with Ce 74.3% with Fe	# 40.5%	35 min	UV	[64]
Rhodamine B	Ag	Solvothermal	100%	15%	60 min	Visible	[65]
Amaranth	Ag	Chemical, mixed by sonication	100% + 100% -	* 99.2% + * 99.96% -	4 h + 3 h -	UV Solar	[66]
Orange II	Ag	Sol-gel, mixed by stirring	90% + 40% ++	90% + 5% ++	2 h	UV Visible	[67]
Black 5			100% + 30% ++	80% + 10% ++			
Methyl orange	Ag	Sol-gel and thermal	97.67%	72.53%	3 h	Solar	[68]
Rhodamine B	Ag	Chemical, microwave assisted	99%	88%	3 h	UV	[69]
Indigo carmine	Gd	Hummers’ method, sol-gel	97%	19%	210 min	Visible	[70]

+ Under UV radiation; ++ under visible light; - under sunlight; * values for binary composites TiO_2_ + metal; # values for binary composites TiO_2_+GO; WD without data.

An interesting study by Roşu and co-authors [66] monitored the degradation of amaranth under UV, intense sunlight and day light for 4 h. They tested a TiO_2_-Ag (TA) composite and a ternary GO/TiO_2_-Ag (TA-GO) composite; besides, TA-GO was annealed at 300 °C to reduce graphene oxide (TA-GR) and a certain amount of TA-GR was thermally treated at 550 °C in an H_2_/Ar-obtaining TA-GT sample. The photocatalytic activity of TA-GO, TA-GR and TA-GT was investigated and compared with that of TA sample and the results of the degradation of amaranth can be observed in Figure 4b–d for the three different types of radiation: UV, intense sunlight, and day light, respectively. We can highlight the fact that in all the experiments, the ternary composites, in any of its variants, outperform TiO_2_-Ag composites, proving the effectiveness of introducing graphene oxide in the nanosystem, confirming the results of Ben Saber, as discussed above. Moreover, the ternary composites perform better under intense sunlight compared to UV radiation, achieving a complete degradation of the dye after 3 h; see Figure 4b,c. Finally, although full degradation of the dye is not possible under day light, see Figure 4d, it is noteworthy that the ternary composite TA-GR (reduced graphene oxide, TiO_2_, Ag) achieves ~95% degradation after 4 h of radiation, holding promise for a material that allows dye degradation at average solar irradiation. Later, in 2017, the same group [61] fabricated a GO/Pt/TiO_2_ nanocomposite and tested the UV and solar photodegradation of three different dyes (amaranth, sunset yellow and tartrazine). After three hours of irradiation, they proved better degradation efficiencies under solar irradiation compared to UV, being able to degrade 99.56%, 99.15% and 96.23% of amaranth, sunset yellow and tartrazine, respectively. A descriptive image of these experiments is shown in Figure 4e, where the decolorization of the dyes after irradiation is evident to the naked eye. Hsieh et al. [62] and Al-Mamun et al. [68] also tested their metallic nanocomposites (Pt and Ag, respectively) under solar irradiation, finding degradation efficiencies close to 100% for acid orange 7 and methyl orange; see Table 1. It is very interesting that many research groups have carried out dye photodegradation experiments under visible or solar irradiation, proving the efficiency of GO and metallic nanoparticles to red shift the band gap energy of TiO_2_ to the visible range, overcoming one of the disadvantages of pure titania photocatalyst and rendering possible the utilization of solar radiation for the photodegradation processes of waste waters.

### 3.2. X = Semiconductors

The synergetic behavior of GO and TiO_2_ with semiconductor nanoparticles has been explored for the photodegradation of methylene blue, methyl orange, rhodamine B, reactive blue 19, congo red, acid blue 25, crystal violet and amido black-10B. Research groups have investigated the photocatalytic activity of ternary compounds introducing semiconductive materials such as ZnO [71,72,73], SnO_2_ [74], BiVO_4_ [75], BiOCl [76], Nb_2_O_5_ [77], Ag_3_PO_4_ [78,79], AgFeO_2_ [80], Cr_2_S_3_ [81], g-C_3_N_4_ [82], ZnS [83,84,85,86]; a compendium of these reports is given in Table 2.

**Figure 5:**
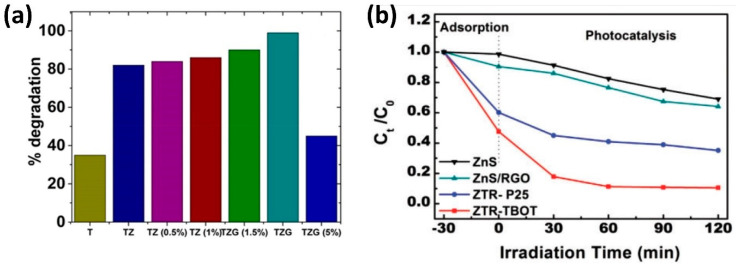
Photocatalytic degradation of methylene blue by ternary composites of ZnS, graphene oxide and titania. (**a**) Photocatalytic degradation under sunlight for ternary composites of TiO_2_/ZnS/GO with different proportions of GO (adapted with permission from [86], Copyright 2020 American Chemical Society). (**b**) Comparison of the photocatalytic degradation of MB under visible light (Xe lamp) by pure ZnS, ZnS/rGO binary composite and ternary composites ZnS/rGO/TiO_2_ for two types of titania nanoparticles (P25 and synthesized from tetrabutyl titanate) (reproduced from Sage Journals under the Creative Commons Attribution 4.0 license from [84]).

Several interesting remarks can be made from these works, the first one is the use of radiation in the visible range in most cases (Maarisetty and co-authors [86] used solar radiation in their report, Potle et al. [73] and Shehzad et al. [80] used UV-Visible radiation), evidencing the modification of the band gap to achieve visible radiation-active photocatalysts. The second remark is the high dye degradation activity, for most cases above 90%, only Raliya and colleagues [72] obtained a degradation efficiency of 44.2%; however, in all cases, the ternary composites yielded better performance than pure titania or binary composites, as can be confirmed in Table 2. This tendency can be observed in Figure 5a,b where the results of the efficacy of GO/ZnS/TiO_2_ ternary photocatalysts for the degradation of methylene blue are depicted from the reports of Maarisetty et al. [86] and Qin et al. [84], respectively. In Figure 5a, we observe an optimum performance (100% degradation) of the ternary composite GO/ZnS/TiO_2_ with 2.5 wt% of GO (TZG), compared to pure titania (T) and binary ZnS/TiO_2_ composites (TZ). An interesting (and recurrent) phenomenon is the fact that for the TZG material with concentration above 2.5 wt% of GO, the degradation efficiency depletes, showing the worst behavior of the different TZG samples. This effect is observed in other publications, evidencing that increasing the amount of GO or the third component beyond a certain concentration not only does not improve the degradation rate but diminishes it. In Figure 5b, the superiority of the GO/ZnS/TiO_2_ ternary composite (ZTR-TBOT) against pure ZnS and ZnS/rGO binary composites is evidenced. Finally, the third remark pertains to the reduced times required for degradation in most cases; in the study by Jing and coworkers [76], a rapid degradation of Rhodamine B was achieved using a ternary composite of GO/BiOCl/TiO_2_ under visible radiation, degrading above 90% of the dye in only 5 min.

The synergistic effect of two different photocatalysts in contact with graphene oxide prevents the recombination of electron-hole pairs and reduces the band gap energy, boosting the capacity of redox reactions at the surface of the composite triggered in the visible range of the electromagnetic spectrum. Furthermore, the reusability of the ternary nanocomposites was explored by Zhu et al. [75], Al Kausor et al. [79] and Maarisetty et al. [86]. The material GO/TiO_2_/ZnS was tested for four cycles [86], with washing procedures with deionized water and ethanol followed by drying after each cycle, proving high degradation efficiency (~90% after the fourth cycle). The ternary composite using Ag_3_PO_4_ demonstrated degradation efficiency close to 90% at the fifth cycle of reusability [79]; while BiVO_4_ composite also showed high efficiency after five cycles of reusability [75], provided washing and drying procedures are performed after each run. Although not stated in the later articles, the recovery and reusability of the photocatalytic materials relies on precipitation by centrifugation procedures, mass recovery yield is a parameter seldom discussed.

### 3.3. X = Magnetic Nanomaterials

In order to foster the reusability and manipulation of TiO_2_/GO materials, many research groups have incorporated magnetic nanomaterials into the nanocomposites. As described above, binary composites constituted of TiO_2_ and GO have superior performance compared to pure TiO_2_, by introducing magnetic nanoparticles as third constituent, the composites acquire the ability to be collected by external magnetic fields, promoting their reusability. We have mainly found reports using Fe_3_O_4_ (magnetite) nanoparticles, although other magnetic oxides have been tested. Table 3 summarizes the main characteristics of ternary magnetic nanocomposites.

#### 3.3.1. GO/TiO_2_/Magnetite

**Figure 6:**
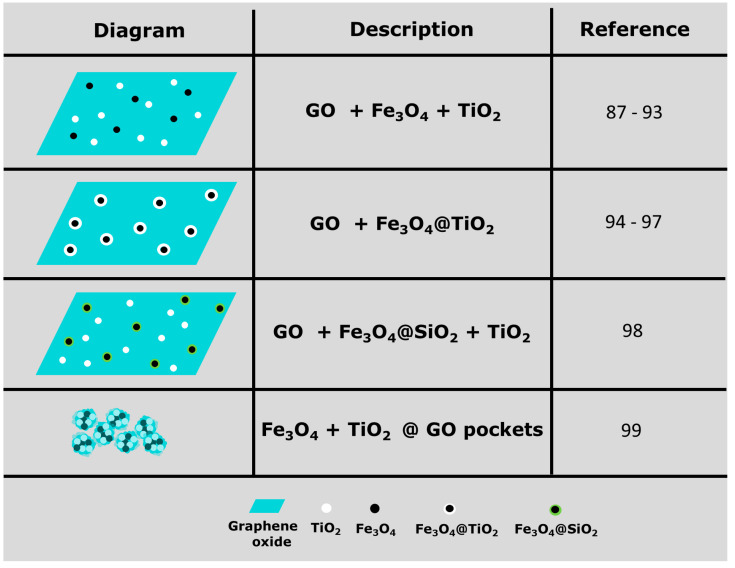
Schematic representations of the different configurations reported in the literature for ternary nanocomposites of graphene oxide, magnetite nanoparticles (Fe_3_O_4_) and titania nanoparticles (TiO_2_) [87,88,89,90,91,92,93,94,95,96,97,98,99].

Ternary magnetic photocatalytic GO composites have been investigated for the photodegradation of dyes (methylene blue, rhodamine B, methyl orange, crystal violet, tartrazine and malachite green). Although the most common configuration of the nanocomposite consists of graphene oxide layers decorated with magnetite (Fe_3_O_4_) and titania (TiO_2_) nanoparticles [87,88,89,90,91,92,93]; other morphologies have been explored, such as: core/shell Fe_3_O_4_@TiO_2_ nanoparticles on graphene oxide [94,95,96,97]; core/shell Fe_3_O_4_@SiO_2_ + TiO_2_ nanoparticles on graphene oxide [98], and graphene sheets wrapping Fe_3_O_4_ and TiO_2_ nanoparticles in a sort of nanocapsule [99]; a schematic summary of these morphologies is presented in Figure 6. Coprecipitation is a widely used technique to synthesize inorganic nanoparticles; two or more water soluble salts react in aqueous solution to form a water insoluble salt that precipitates and forms nanoparticles. In the case of Fe_3_O_4_, hydrated ferrous chloride (FeCl_2_∙4H_2_O) and ferric chloride (FeCl_3_∙6H_2_O) undergo oxidative reaction following deprotonation in aqueous solution in the presence of NH_3_∙H_2_O (or NH_4_OH) as the precipitating agent. When the concentration of Fe_3_O_4_ product in the solution increases above the solubility limit, the product precipitates by nucleation in the liquid phase. The growth of Fe_3_O_4_ crystals continues with precipitation reaction on the nucleating surfaces. Finally, the small particles undergo agglomeration via Ostwald ripening and form Fe_3_O_4_ nanoparticles [45].

**Figure 7:**
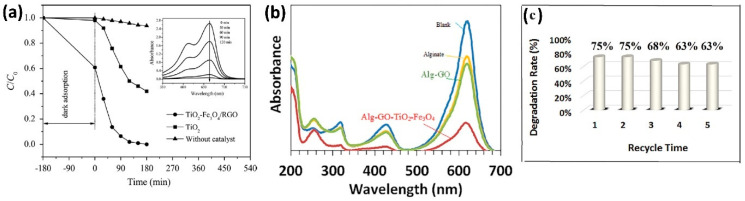
Photocatalytic degradation of (**a**) methylene blue, (**b**,**c**) malachite green by ternary magnetic composites based on GO/Fe_3_O_4_/TiO_2_. (**a**) Comparison between TiO_2_ and ternary composite photodegradation under visible light radiation; (**b**) sonophotocatalytic degradation under UV radiation; (**c**) reusability of ternary composite tested in (**b**). Image credits (**a**) reproduced with permission from Elsevier, license number 5661610809492, from [95]; (**b**,**c**) reproduced with permission from Taylor and Francis, license 5661630124794, from [93].

The advantages of these ternary nanocomposites are, on one hand, the shift of the band gap energy from the UV for TiO_2_ to the visible range of the electromagnetic spectrum, and on the other hand, the easiness on the separation of the catalyst by means of a magnetic field. Li et al. [95] characterized their materials by UV-Vis diffuse reflectance spectra and determined the energies for pure TiO_2_ and their TiO_2_–Fe_3_O_4_/RGO composite to be 3.12 and 2.12 eV, respectively, demonstrating a considerable redshift of the visible region, rendering the material suitable for degradation using solar irradiation; the efficiency of the ternary composite vs pure TiO_2_ is evident in Figure 7a. Nada and co-workers [90] report a band gap of 2.42 eV for the ternary composite that showed the best performance in the photocatalytic degradation of tartrazine (95.5% degradation). These results agree with the conclusion of Sedghi and co-authors [89]; in their study, TiO_2_ displays a response in the range of 200–400 nm (UV region), as expected, while the absorption band of the TiO_2_/magnetic pGO nanocomposites shifted to the visible light region.

The other advantage of these magnetic GO-TiO_2_ photocatalysts is their facile separation from the aqueous medium by a magnet once the photodegradation has been carried out, this advantageous characteristic allows the reusability of the materials, being able to perform several cycles of photodegradation as tested by Li et al. [95], who observed a mild drop in degradation efficiency (from 99.5% to 92.1%) after five cycles, which indicates a high stability of the photocatalyst nanocomposite. Rashidzadeh et al. [93], photodegraded malachite green (see Figure 7b) and tested the material for five cycles (see Figure 7c); their findings reveal only a small decrease in photocatalytic performance. Other groups agree with this conclusion, such is the case of Linley et al. [98], Sedghi et al. [89], Piranshahi et al. [96], Nada et al. [90], Nadimi et al. [92], evidencing the high stability of these composites. Moreover, Linley and co-workers [98] demonstrated that the activity of the catalyst can be regenerated by suspending the particles in water for 16 h and exposing them to UV-A radiation; this cleanses the active sites on the catalyst surface from unreacted methylene blue or degradation by-products.

#### 3.3.2. Other Magnetic Materials

Ternary magnetic materials have also been synthesized with other magnetic metallic oxides, such as cobalt ferrite (CoFe_2_O_4_) [100,101], magnesium ferrite (MgFe_2_O_4_) [102] and nickel ferrite (NiFe_2_O_4_) [103,104] and tested for the photodegradation of dyes. Such nanocomposites also exhibit good dye photodegradation and reusability.

### 3.4. X = Other

Literature reports on ternary GO/TiO_2_ composites in which the third component could not be classified as metal, semiconductor or magnetic compound, are listed in Table 4. We found that GO and TiO_2_ nanoparticles have been coupled with hemin [105], SiO_2_ [106,107], polyaniline [108], and hydroxyapatite nanoparticles [109]; these ternary nanocomposites have been evaluated as photocatalysts for the successful removal of dyes. The reported degradation efficiencies are high (the lowest reported is 84% for methylene blue in a GO/SiO_2_/TiO_2_ composite [107]), proving good photocatalytic performances. Moreover, even though no metals or semiconductors are being tested, the validation of the use of the ternary composites in the visible range is proven since most reports use visible radiation. It is noteworthy the fact that in all reports, the ternary composites outperform pure materials and binary nanocomposites, a drawback of these tested materials is the long times involved in the degradation experiments—all experiments considered times of one hour or longer periods, up to 7 h in the case of Ahmad and co-authors [107].

## 4. Conclusions

The synergistic interaction of GO and TiO_2_ has been tested along with metals, semiconductors and magnetic nanomaterials to enhance titania’s photocatalytic performance on dye degradation. In the literature, reports on GO/TiO_2_/semiconductors ternary nanocomposites outnumber the reports of ternary composites coupled with metals or Fe_3_O_4_ (magnetite). The majority of the reports use visible light (a few use solar light) which evidences the success of red shifting the photocatalytic activity of pure anatase phase titania by combining graphene oxide and the third components. It is noteworthy that many reports evaluate the recyclability of their materials, proving a paradigm change towards disposability; researchers are conscious of the necessity of reusing the photocatalytic materials to embrace a sustainable approach to wastewater management.

## 5. Challenges and Perspectives

Beyond the positive results of these ternary composites for the photoreduction of dyes, a critical discussion needs to be raised regarding the challenge of determining the proportions of each component to rightly tune the optimal photocatalytic behavior. Many studies report the fact that when testing different proportions of the ternary composites, increasing the amount of GO or the third component does not necessarily yield an increase in dye degradation efficiency; adverse effects are observed. Thus, the optimal “recipe” for a particular combination of constituents and a particular synthesis method is obtained experimentally, however, up to now, there is no rule to be applied universally. Systematic studies that provide insight into this matter are pertinent.

Another challenge that directly relates to the point raised above is the heterogeneity of the nanomaterials used in the different studies. GO’s characteristics depend on the synthesis method, whether it is reduced or not, how it was stored, which functional groups are predominant, and sheet sizes. Regarding TiO_2_, many studies rely on commercial P25 samples, however, an important number of contributions synthesize the TiO_2_ nanomaterials through chemical synthesis, hence, the morphologies, sizes, crystallinity, and ratio of anatase to rutile are not constant. This heterogeneity might have an effect on the reproducibility of results. Another challenge lies in the scalability of the production methods and on the high energy consumption involved in them; most synthesis processes rely on wet chemistry, hand in hand with washing cycles, long time drying processes along with thermal treatments; for lab-sized batches this is viable, but having in mind large quantities for treatment of wastewaters, these processes need to be rethought with an approach toward sustainability.

## Figures and Tables

**Table 2:** Summary of literature review for the photocatalytic degradation of dyes using ternary composites of GO, semiconductors and titania (TiO_2_) nanoparticles.

Dye Degraded	Catalyst		Catalyst Efficiency		Degradation Time	Radiation Type	Reference
	Semi Conductor	Composite Synthesis Process	Ternary Nanocomposite	TiO_2_			
Methylene blue	ZnO	Hummers’ method, solvothermal	92%	47%	120 min	Visible	[71]
Methyl orange	ZnO	Hummers’ method, hydrothermal, sol-gel and mixed by sonication	44.2%	28%	120 min	Visible	[72]
Crystal violet	ZnO	Hummers’ method, sonochemical	89.63%	WD	20 min	UV	[73]
Congo red	SnO_2_	Hummers’ method, solvothermal	98%	26%	70 min	Solar	[74]
Methylene blue			96%	21%	60 min		
Reactive Blue 19	BiVO_4_	Hummers’ method, hydrothermal	95.87%	* 35.6%	90 min	Visible	[75]
Methylene blue	BiOCl	Hummers’ method, solvothermal	98.2%	# 72.5%	40 min	Visible	[76]
Amido Black-10B			96%	WD	10 min		
Methyl orange			98.3%	WD	15 min		
Rhodamine B			90.5%	WD	5 min		
Methylene blue	Nb_2_O_5_	Hydrothermal	97%	35%	4 h	Visible	[77]
Methyl orange			93%	15%			
Methylene orange	Ag_3_PO_4_	Modified Hummers’ method	80%	WD	120 min	Visible	[78]
Acid Blue 25	Ag_3_PO_4_	Hummers’ method, chemical	97%	25%	45 min	Visible	[79]
Methylene blue	AgFeO_2_	Chemical and reflux	100%	98%	30 min	UV-Vis	[80]
Methylene blue	Cr_2_S_3_	Modified Hummers’ method, sol-gel	98.3%	60%	120 min	Visible	[81]
Rhodamine B			96.6%	64%			
Methyl orange			86.3%	50%			
Methylene blue	g-C_3_N_4_	Sol-gel, solvothermal, calcination	98.84%	38%	120 min	Visible	[82]
Methylene blue	ZnS	Modified Hummers’ method, sol-gel	90.1%	$ 10%	150 min	Visible	[83]
Methylene blue	ZnS	Solvothermal	90%	□ 30%	120 min	Visible	[84]
Crystal violet	ZnS	Modified Hummers’ method, sonochemical	97.02%	WD	50 min	Visible	[85]
Methylene blue	ZnS	Modified Hummers’ method, solvothermal	100%	35%	WD	Solar	[86]

* Value for the activity of BiVO_4_; # value for the activity of composite rGO/BiOCl; $ value for the activity of composite ZnS/graphene; □ value for the activity of ZnS; WD without data.

**Table 3:** Summary of literature review for the photocatalytic degradation of dyes using ternary composites of GO, magnetic nanomaterials and titania (TiO_2_) nanoparticles.

Dye Degraded	Catalyst		Catalyst Efficiency		Degradation Time (min)	Radiation Type	Reusability	Reference
	Material	Composite Synthesis Process	Composite	TiO_2_				
Rh B	GO/TiO_2_/Fe_3_O_4_	Hummers’ method, hydrolysis, coprecipitation	100% *	47.5%	25	UV	5 cycles	[87]
Methyl orange	GO/Fe_3_O_4_/TiO_2_	Impregnation, mixed by sonication	100% *	100%	30	Visible	3 cycles **	[88]
Rh B	GO/Fe_3_O_4_/TiO_2_	Hummers’ method, coprecipitation, hydrolysis, mix and reflux	99%	75%	20	Visible	10 cycles	[89]
Tartrazine	GO/Fe_3_O_4_/TiO_2_	Hummers’ method, coprecipitation, hydrolysis, mixed by sonication	95.5%	10%	210	Visible	4 cycles	[90]
Methylene blue	GO/Fe_3_O_4_/TiO_2_	Hummers’ method, hydrothermal	88.11% + 90.52% ++	97.61 + 45% ++	55	UV Visible	WD	[91]
Methylene blue	GO/Fe_3_O_4_/TiO_2_	Hummers’ method, sonochemical, hydrolysis	82% + 76% ++	72.5% + 45% ++	90	UV Visible	4 cycles	[92]
Malachite green	GO/Fe_3_O_4_/TiO_2_	Hummers’ method, coprecipitation, sol-gel, mixed by sonication	75%	WD	60	UV ∙	5 cycles	[93]
Methyl orange	Fe_3_O_4_@TiO_2_/rGO	Solvothermal, vapor-thermal, hydrothermal	83.8%	33%	50	UV	WD	[94]
Methylene blue	Fe_3_O_4_@TiO_2_/rGO	Hummers’ method, chemical, hydrolysis, hydrothermal	99.5%	58%	120	Visible	5 cycles	[95]
Crystal violet	GO/Fe_3_O_4_@TiO_2_	Hummers’ method, coprecipitation, hydrolysis	98.5%	WD	60	UV	4 cycles	[96]
Rh B	Fe_3_O_4_@TiO_2_/GO	Hummers’ method, hydrolysis, mixed by stirring	52.9%	27.2%	60	Solar	WD	[97]
Methylene blue	Fe_3_O_4_@SiO_2_/TiO_2_/GO	Hydrothermal, Stöber reaction, hydrothermal	92% *	90%	75	UV	5 cycles	[98]
Methyl orange	Fe_3_O_4_/TiO_2_@GO	Hummers’ method, mixed by ultrasonication, aerolized	98%	75%	60	UV	5 cycles	[99]
Methyl orange	TiO_2_/CoFe_2_O_4_/rGO	Hummers’ method, chemical oxidation, reflux	100%	# 10%	75	Visible	5 cycles	[100]
Methylene blue	TiO_2_/CoFe_2_O_4_/GO	Hummers’ method, hydrothermal	99%	& 15%	300	Visible	3 cycles	[101]
Methylene blue	GO/MgFe_2_O_4_/TiO_2_	Modified Hummers’ method, sol-gel, mixed by sonication	99% + 99% ++	95% + 60% ++	120	UV Visible	WD	[102]
Methylene blue	TiO_2_/NiFe_2_O_4_/rGO	Hummers’ method, hydrothermal, mixed by sonication	87% + 71% ++	74% + 42% ++	90	UV Visible	4 cycles	[103]
Methylene blue	rGO/TiO_2_/NiFe_2_O_4_	Hummers’ method, hydrothermal	100% + 100% ++	98% + 5% ++	105	UV Visible	10 cycles	[104]

* Not stated in the publication, estimated from the articles’ plots; ** in acidic conditions; + under UV radiation; ++ under visible light; Rh B stands for Rhodamine B; GtO stands for graphite oxide; **∙** Sonophotocatalysis; WD without data; # value for CoFe_2_O_4_; & value for TiO_2_/GO binary composite.

**Table 4:** Summary of the literature review for the photocatalytic degradation of dyes using ternary composites of GO, titania (TiO_2_) nanoparticles and heterogenous third components.

Dye Degraded	Catalyst		Catalyst Efficiency		Degradation Time	Radiation Type	Reference
	Third Component	Composite Synthesis Process	Ternary Nanocomposite	TiO_2_			
Rhodamine B	Hemin	Hummers’ method, sol-gel	100%	5%	1 h	UV	[105]]
Methylene blue	SiO_2_	Improved Hummers’ method, sol-gel	84.82%	55%	3 h	Visible	[106]
Crystal violet			85.45%	65%			
Methylene blue	SiO_2_	Sol-gel	99%	WD	7 h	Solar	[107]
Rose Bengal	Polyaniline	Hummers’ method, chemical	97%	25%	3 h	Visible	[108]
Thymol blue			96%	20%			
Methylene blue	Hydroxyapatite	Hummers’ method, stirring and sonication	98%	22%	1 h	UV	[109]

WD without data.
